# Validity of Body Image Pictogram to Determine Overweight/Obesity in Adults from Less Developed Populations: Results From Pars Cohort Study

**DOI:** 10.34172/aim.2022.123

**Published:** 2022-12-01

**Authors:** Somayeh Bazdar, Mohammad Hossein Sharifi, Hossein Puostchi, Abdullah Gandomkar, Reza Malekzadeh, Fatemeh Malekzadeh, Hossein Molavi Vardanjani

**Affiliations:** ^1^MPH Department, School of Medicine, Shiraz University of Medical Sciences, Shiraz, Iran; ^2^Research Center for Traditional Medicine and History of Medicine, Shiraz University of Medical Sciences, Shiraz, Iran; ^3^Liver, Pancreatic, and Biliary Diseases Research Center, Digestive Disease Research Institute, Tehran University of Medical Sciences, Tehran, Iran; ^4^Non-Communicable Disease Research Center, Shiraz University of Medical Sciences, Shiraz, Iran; ^5^Digestive Diseases Research Center, Digestive Diseases Research Institute, Shariati Hospital, Tehran University of Medical Sciences, Tehran, Iran; ^6^MPH Department, School of Medicine, Research Center for Traditional Medicine and History of Medicine, Shiraz University of Medical Sciences, Shiraz, Iran

**Keywords:** Body image pictogram, Ethnicity, Obesity, Pars Cohort Study, Socio-economic status, Validity

## Abstract

**Background:**

Despite the evidence for validity of body image pictogram (BIP) to discriminate overweight, obese, and normal individuals, there is little evidence on the probable effect of socio-demographic variables on its validity. To investigate the effects of socioeconomic status (SES), age, ethnicity, and educational level on the validity of BIP to discriminate normal weight, overweight, and obese people.

**Methods:**

We used the Pars Cohort Study (PCS) data. Stunkard’s BIP score was used as test measure. Participants were classified as normal (body mass index [BMI]<25), overweight (BMI=25 to 29.9), and obese (BMI≥29.9) based on their BMI (kg/m^2^ ). Area under curve (AUC) and its 95% CI were estimated and compared. Optimal cutoff points and their sensitivity, specificity, and likelihood ratio were reported.

**Results:**

A total of 9232 participants with a female/male ratio of 1.03 were included. The prevalence of overweight and obesity was 37.4% and 18.2%, respectively. Regardless of socio-demographic levels, the optimal cut-points to discriminate normal BMI from overweight, and overweight from obese participants were BIP score of four and five, respectively. Estimated AUC correlated with ethnicity (*P*<0.001) for both genders, and with SES for females (*P*<0.05).

**Conclusion:**

Although BIP may be a valid measure to categorize the general adult population into normal, overweight and obese, its validity depends on SES and ethnicity. BIP may be available as a proxy measure for BMI categories in socio-demographically homogeneous populations but not in heterogeneous populations.

## Introduction

 The obesity epidemic is among the most important causes of the growing global burden of non-communicable diseases.^1^ The epidemic is also one of the most challenging public health problems, especially in developing countries.^2^ In addition to obesity, there is a huge body of evidence that shows overweight as a significant risk factor for non-communicable diseases.^3,4^

 A set of predefined cut-off values of body mass index (BMI) are the current gold standard to determine if a person is obese/overweight or not.^5^ Accordingly, obesity is defined as a BMI of 30 (kg/m^2^) or more, based on the World Health Organization (WHO) standards. Overweight means that a person has a BMI of 25 (kg/m^2^) up to less than 30 (kg/m^2^).^6^ These cut-off values have been defined based on their validity to predict cardio-metabolic events in large scale cohort studies.^6^ Therefore, in most epidemiological studies, aiming to predict cardio-metabolic events, it is mostly needed to determine if the person is obese/overweight or not, but not to determine their precise BMI value.^7^ This is the case in most of the published studies, where weight and height are measured and then BMI is calculated, and based on the calculated BMI, the study participants are classified into normal/overweight/obese subgroups.^7^

 On the other hand, in large-scale epidemiological studies, measurement of height and weight needs calibrated equipment, and trained personnel which may not be available in low-resource settings.^8^ In addition, these measurements are possible to be conducted in non-retrospective studies in which participants are asked to be present in the study setting for the study measurements.^9^ Consider a large-scale epidemiological study which is conducted in a low-resource setting, in which questionnaires are mailed to a sample of less literate adults. How can the study investigators measure the participants’ obesity/overweight? They would have to rely on self-reported heights and weights, or use alternative methods. Keshtkar et al^10^ recommended that Stunkard’s body image pictogram (BIP) is an alternative method to measure overweight/obesity in adults.

 While some large-scale prospective cohort studies, i.e., Nurses’ Health Study (NHS), have used BIP as a measure of obesity/overweight,^11^ it was originally introduced and has been widely used in studies as a tool to determine body image.^12,13^ Limited data are available on the validity of BIP as a method to measure obesity/overweight.^10^ Although the BIP is not designed to measure BMI, it may be applicable to determine overweight/obesity as an alternative method in some settings.

 Some authors have argued that the participants’ body image may be associated with the participants’ characteristics such as gender, age, ethnicity, desired body shape, socioeconomic status (SES), educational level, and also living conditions.^14,15^ Differential misclassification across population subgroups may results in biased results in epidemiological studies.^16^ Therefore, we need to investigate the validity of BIP across population subgroups.

 Hypothesizing that SES, age, gender, ethnicity and education may be some of the most important determinants of body perception, we aimed to investigate the effect of SES, age, gender, ethnicity and education on the validity of self-reporting BIP to identify normal weight, overweight, and obese participants in a less developed region. In this study, we have a great opportunity to use baseline data from a well-established large-scale population-based cohort study in this region, i.e., Pars Cohort Study (PCS).

## Materials and Methods

###  Study Setting and Participants

 This study is a cross-sectional validity study, in which we used baseline data of PCS, a study that launched in 2014. PCS is an ongoing large-scale population-based cohort in Valashahr district, Iran. The catchment area of PCS, Valashahr district, is a semi-urban district located in southwestern Iran populated by around 40 000 people, mostly from Persian or Turk (Ghashghaei) ethnicities. The cohort has been designed to investigate the epidemiology of the risk factors of non-communicable diseases in a less-developed region. More details on the PCS methodology are previously published.^17^

 In brief, the inhabitants of Valashahr aged 40-75 years (n = 9721) were invited to participate in PCS, and a total of 9264 were enrolled into the PCS baseline measurements. None of them were excluded except for those who were not interested in participating in the study. A comprehensive face-to-face interview based on a standardized structured questionnaire, physical examinations, anthropometric measurements, and biological sampling were performed at the PCS baseline phase. The study measurements were made in the PCS center by experienced nurses or physicians using calibrated equipment. More than 200 variables were measured during the baseline phase. More details about the measured variables have been presented elsewhere.^17^

###  Variables Used in this Study

**Figure 1:**
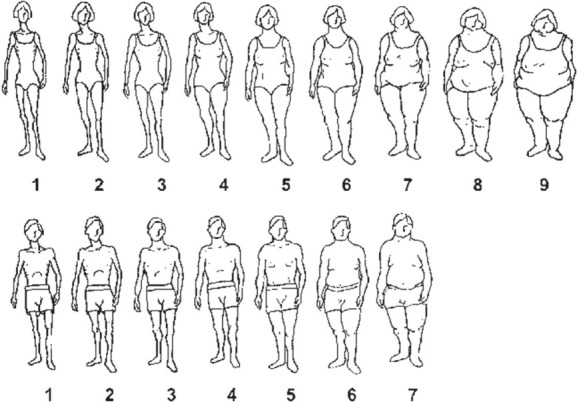
Body Shape Pictograms Used in the Pars Cohort Study

 We used data on height (cm); weight (kg); age (younger than 50 years, 50 to 59 years, and 60 years and older); gender; education (illiterate, less than diploma, and more than diploma); ethnicity (Persian, Turk, and others); current marital status (married, single); socio-economic assets; and body shape. Data on body shape was collected using an adopted version of gender-specific body shape pictograms originally designed and introduced by Stunkard et al.^12^ These self-report BIPs included seven and nine body shapes for males and females, respectively (Figure 1).

 Using the participants’ height and weight, we calculated BMI and categorized it based on the cut-points recommended by the WHO.^6^ Accordingly, participants were categorized into three BMI categories including underweight or normal weight (BMI of less than 24.9 kg/m^2^), overweight (BMI of 25 to 29.9 kg/m^2^), and obese (BMI of more than 29.9 kg/m^2^).

 Socio-economic assets were analyzed applying multiple correspondence analysis (MCA). The participants’ SES was determined based on the quartiles of the estimated latent factor by MCA. Accordingly, participants were categorized into four relatively equal groups including high, high-middle, middle-low, and low SES. These groups were not completely equal because of ties in ranks based on the estimated latent variable.

###  Statistical Methods

 Data was described using absolute and relative frequencies. No imputation technique was applied on missing data because of ignorable rates (less than 1% of data was missing). Prevalence of obesity and overweight was estimated based on binomial distribution. Age-standardized prevalence of overweight and obesity and their 95% confidence interval (CI) were estimated, considering the 2000–2025 world standard population.

 The conceptual framework of the statistical analyses applied in the study is presented in additional file 1. Two dummy variables were generated based on the BMI categories. One of them classified the study participants into two groups (BMI of less than 25 kg/m^2^ VS BMI of 25 kg/m^2^ and more), and the second classified the study participants into two groups (BMI of less than 29.9 kg/m^2^ VS BMI of 29.9 kg/m^2^ and more). These variables were considered as gold standards. Two series of analysis were done for each of these gold standards. BIP scores were considered as test measure. The discriminative ability of BIP was assessed using receiver operating characteristic (ROC) curve. Area under curve (AUC) and its 95% CI were estimated and compared across different subgroups regarding SES, age, ethnicity, and education. Sensitivity and specificity and their 95% CI were estimated for each of BIP scores. Comparing calculated Youden Indies for different scores, the scores with the highest possible values for both sensitivity and specificity were reported as the optimal cut-point. AUC, sensitivity, and specificity and their 95% CI were reported for optimal cut-points. Data analysis was done separately for males and females. Data analysis was done using the Stata software (College Station, TX: Stata Corp LLC). A two-sided *P* value of less than 0.05 was considered statistically significant.

## Results

 A total number of 9232 participants, including 4265 (46.2%) males and 4967 (53.8%) females, with a mean age of 52.6 ± 9.7 were analyzed. The overall prevalence of overweight and obesity was 37.4% (36.4% to 38.4%) and 18.2% (17.4% to 19.0%), respectively. Age-standardized prevalence was 36.4% (35.4% to 37.5%) for overweight, and 17.2% (16.3% to 18.0%) for obesity. The age-standardized prevalence of obesity and overweight was 8.6% (7.3% to 9.2%) and 32.2% (31.4% to 33.3%) for males, and 24.9% (23.8% to 26.2%) and 40.4% (368.2% to 41.54%) for females, respectively. The prevalence of obesity and overweight was significantly different across SES levels (*P* < 0.001; Table 1).

**Table 1:** Characteristics of the Study Participants and Prevalence of Obesity and Overweight Among Adults, Southwestern Iran

Characteristic	**Overall, ** ***n*** ** (%** ^*^ **)**	**Obese, ** ***n*** ** (P%** ^**^ **); (95% CI)**	**Overweight, ** ***n*** ** (P%** ^**^ **); (95% CI)**
Gender			
Male	4265 (46.2)	386 (9.1); (9.0– 9.2)	1427 (33.6); (32.2– 34.3)
Female	4967 (53.8)	12.8 (26.0); (24.8– 27.2)	2010 (40.1); (38.1– 42.0)
Age group			
40–50	4203 (45.5)	824 (19.7); (18.8– 20.5)	1657 (39.6); (38.4– 40.3)
50–59	2798 (30.3)	506 (18.2); (17.1– 19.3)	1047 (37.7); (36.0– 38.8)
60 +	2231 (24.2)	340 (15.3); (14.1– 16.4)	733 (33.0); (31.9– 34.6)
Socio economic status			
Low	2408 (26.1)	338 (14.2); (13.2– 15.6)	762 (31.9); (0.12–0.15)
Low – Middle	2490 (27.0)	372 (15.0); (13.9– 16.0)	913 (36.8); (35.3– 37.6)
Middle – High	2043 (22.1)	425 (20.9); (19.8– 21.2)	763 (37.5); (36.1– 39.2)
High	2291 (24.8)	535 (23.4); (22.0– 24.5)	999 (43.8); (42.9– 44.8)
Educational levels			
Illiterate	4523 (49.0)	842 (18.7); (17.8– 19.9)	1607 (35.8); (35.0– 36.6)
Less than diploma	3691 (40.0)	779 (17.7); (16.1– 18.8)	1432 (39.0); (37.9– 40.9)
More than diploma	1012 (11.0)	48 (17.3); (16.0– 18.4)	397 (38.4); (37.3– 40.0)
Marital status			
Married	8186 (88.7)	1473 (18.1); (17.0– 19.1)	3025 (37.1); (36.2– 38.2)
Single	1043 (11.3)	196 (18.9); (18.0– 19.7)	411 (39.7); (18.7– 40.6)
Ethnicity			
Persian	5196 (56.3)	1053 (20.4); (19.3– 21.3)	1967 (38.0); (36.9– 39.1)
Turk	3585 (38.8)	518 (14.5); (13.3– 15.4)	1293 (36.3); (35.1– 37.5)
Others	451 (4.9)	99 (22.1); (21.0– 23.2)	177 (39.5); (38.4– 4.6)

^*^Percentage = number of participants in each stratum divided by the study total sample size. ^**^Prevalence (%) = number of participants in each stratum who suffering from obesity/overweight divided by the number of participants in the same stratum.

**Figure 2:**
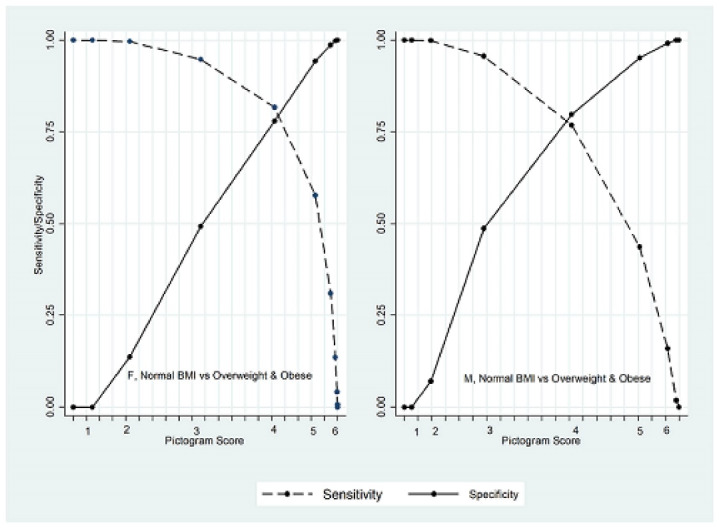
Sensitivity and Specificity of Different Pictogram Scores to Discriminate Normal BMI From Obesity and Overweight by Gender

 The optimal cut-point to discriminate normal BMI from overweight and obese was at the BIP score of 4 for males (AUC = 85.0, 95% CI: 83.9, 86.1) and females (AUC = 87.1; 95% CI: 86.1, 88.0). Figure 2 shows the sensitivity and specificity of different BIP scores to discriminate normal BMI from the overweight and obese population.

 The optimal cut-point to discriminate normal weight from overweight and obese was at 4 in all SES categories in both genders. Comparison of estimated AUC across different SES categories showed that there was a significant difference in females (*P* value = 0.0148) but not among males (*P* value = 0.707; Table 2).

**Table 2:** Discrimination of Normal BMI from Overweight and Obesity According to SES Status Divided by Gender

**Gender/SES**	**TP+FN**	**FP+TN**	**AUC% (95% CI)**	**Cut**	**Sen.% (95% CI)**	**Spe.% (95% CI)**	**Likelihood Ratio+**
**Male**							
Overall	1857	2408	85.0 (83.9, 86.1)	4	76.9 (74.9, 78.8)	79.7 (77.8, 81.1)	3.6
Low	302	669	83.5 (80.9, 86.1)	4	71.9 (66.4, 76.9)	79.7 (76.4, 82.7)	3.5
Low-middle	426	665	84.5 (82.3, 86.8)	4	71.2 (67.7, 76.4)	82.6 (79.5, 85.3)	4.1
Middle-high	446	568	84.8 (82.6, 87.1)	4	79.6 (75.6, 83.2)	76.9 (73.2, 80.3)	3.4
High	683	506	85.44 (83.4,87.5)	4	80.2 (77.0, 83.2)	78.1 (74.2, 81.6)	3.6
*P* value for AUC comparison = 0.707
**Female**							
Overall	3348	1619	87.1 (86.1, 88.0)	4	81.7 (80.3,83)	77.9 (75.8,79.9)	3.5
Low	825	612	87.0 (85.3, 88.8)	4	79.6 (76.7,82.3)	81.2 (77.9, 84.2)	4.2
Low-middle	889	510	84.0 (82.0, 86.0)	4	76.7 (73.8,79.5)	77.1 (73.2,80.6)	3.3
Middle-high	760	269	88.2 (86.0, 90.4)	4	85.3 (82.5, 87.7)	76.6 (71.1, 81.5)	3.6
High	874	228	88.2 (86.0, 90.4)	4	85.6 (83.1,87.8)	72.8 (66.5, 78.5)	3.1
*P* value for AUC comparison = 0.0148

SES, Socio-economic status; TP, true positive; FN, false negative; FP, false positive; TN, true negative; Sen., sensitivity; Spe., specificity; CI, confidence interval; AUC, area under curve.

 The discriminative ability of BIP to discriminate normal weight individuals from participants with overweight or obesity correlated with ethnicity in males (*P* < 0.001) and females (*P* = 0.020). There was a statistically significant difference between estimated AUC for different ethnicities (Table 3).

**Table 3:** Discrimination of Normal BMI From Overweight and Obesity According to Education, Age Groups and Ethnicity Divided by Gender

**Gender/SES**	**TP+FN**	**FP+TN**	**AUC% (95% CI)**	**Cut**	**Sen.% (95% CI)**	**Spe.% (95% CI)**	**Likelihood Ratio+**
**Male**							
Age group							
< 50 years	883	1,046	84.7 (83.1,86.3)	4	75.1 (72.1, 77.9)	80.4 (77.9, 82.8)	3.8
50-59 years	618	733	85.13 (83.2, 87.1)	4	79.1 (75.7, 82.3)	77.5 (74.3, 80.5)	3.5
59 + years	356	629	85.5 (83.2, 87.9)	4	77.5 (72.8, 81.8)	80.3 (77.0, 83.3)	4.0
*P* value for AUC comparison = 0.843							
Education							
Illiterate	464	870	86.4 (85.2, 87.6)	4	73.5 (69.2, 77.5)	81.0 (78.3, 83.6)	3.6
Up to Diploma	913	933	88.1 (86.4, 89.8)	4	78.0 (75.2, 80.6)	76.3 (73.5, 79.0)	3.8
Diploma & more	479	456	81.5 (70.2, 92.7)	4	78.1 (74.1, 81.7)	76.5 (72.4, 80.4)	3.3
*P* value for AUC comparison = 0.785							
Ethnicity							
Persian	1971	865	87.6 (86.2, 88.9)	4	82.7 (81.0, 84.3)	77.8 (74.9, 80.5)	3.8
Turk	1210	681	82.0 (80.0, 83.9)	4	79.6 (77.2, 81.8)	77.5 (74.2, 80.6)	2.9
Others	167	73	83.8 (78.7, 88.9)	4	85.0 (78.7, 90.1)	83.6 (73.1, 91.2)	6.4
*P* value for AUC comparison < 0.001							
**Female**							
Age group							
< 50 years	1,630	645	88.0 (86.5, 89.4)	4	85.8 (84.0, 87.4)	74.4 (70.9, 77.7)	3.3
50-59 years	978	471	86.7 (84.8, 88.6)	4	81.8 (79.2, 84.2)	77.5 (73.5, 81.2)	3.6
59 + years	740	503	84.9 (82.9, 86.9)	4	72.6 (69.2, 75.8)	82.9 (79.3, 86.1)	4.2
*P* value for AUC comparison = 0.050							
Education							
Illiterate	2038	1151	84.8 (82.7, 86.8)	4	79.0 (77.2, 80.8)	79.7 (77.2, 82.0)	3.8
Up to Diploma	1251	448	85.2 (83.6, 86.7)	4	86.1 (84.1, 88.0)	74.1 (69.8, 78.1)	3.3
Diploma & more	58	19	84.2 (81.8, 86.5)	4	81.0 (68.6, 90.1)	63.2 (38.4, 83.7)	2.1
*P* value for AUC comparison = 0.185							
Ethnicity							
Fars	1096	1264	88.0 (86.7, 89.3)	4	80.8 (78.3, 83.0)	82.0 (79.8, 84.1)	3.7
Turk	647	1047	85.3 (83.7, 87.0)	4	72.2 (68.6, 75.6)	75.5 (72.7, 78.0)	3.5
Others	114	97	89.8 (85.8, 93.9)	4	66.7 (57.2, 75.2)	89.7 (81.9, 94.9)	5.1
*P* value for AUC comparison = 0.020							

SES, Socio-economic status; TP, true positive; FN, false negative; FP, false positive; TN, true negative; Sen., sensitivity; Spe., specificity; CI, confidence interval; AUC, area under curve.

**Figure 3:**
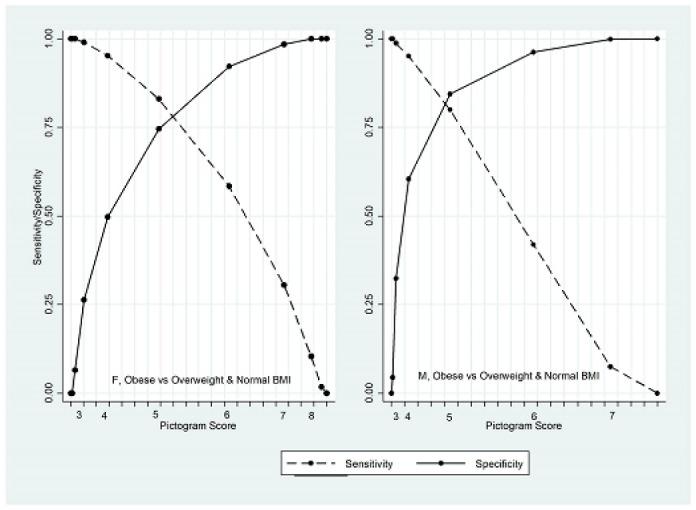
Sensitivity and Specificity of Different Pictogram Scores to Discriminate Obesity From Overweight and Normal BMI by Gender

 The optimal cut-point to discriminate obese from overweight and normal BMI was at the pictogram score 5 for males (AUC = 88.4; 95% CI: 86.9, 89.9) and females (AUC = 86.5; 95% CI: 85.5, 87.6). Figure 3 shows the sensitivity and specificity of different pictogram scores to discriminate obese from the overweight and normal weight population.

 The sensitivity of a pictogram score of 5 to discriminate obesity was estimated at 80.0%. A significance difference was observed between estimated AUC in different SES categories in females (*P* value < 0.001), but not in males (Table 4).

**Table 4:** Discrimination of Obesity from Overweight and Normal BMI According to SES Status and Gender

**Gender/SES**	**TP+FN**	**FP+TN**	**AUC% (95% CI)**	**Cut**	**Sen.% (95% CI)**	**Spe.% (95% CI)**	**Likelihood Ratio+**
**Male**							
Overall	404	3861	88.4 (86.9, 89.9)	5	80.0 (75.7, 83.7)	84.1 (89.2, 85.2)	2.3
Low	56	915	90.1 (86.0, 94.2)	5	82.1 (69.6, 91.9)	87.2 (84.9, 89.3)	2.7
Low-middle	72	1019	86.1 (81.7, 90.5)	4	90.3 (80.4, 95.7)	65.0 (61.7, 67.7)	2.5
Middle-high	112	902	89.5 (86.8, 92.2)	5	83.0 (74.8, 89.5)	86.1 (83.7, 88.3)	2.3
High	164	1025	86.6 (84.2, 89.1)	5	79.9 (72.9, 85.7)	78.8 (76.2, 81.3)	2.0
*P* value for AUC comparison = 0.250							
**Female**							
Overall	1316	3651	86.5 (85.5, 87.6)	5	83.0 (80.8, 85.0)	74.4 (72.9, 75.8)	1.8
Low	289	1148	89.3 (87.4, 91.3)	5	84.1 (79.3, 88.1)	79.6 (77.2, 81.9)	2.2
Low-middle	314	1085	87.5 (85.4, 89.6)	5	84.1 (79.6, 87.9)	77.3 (74.7, 79.8)	2.0
Middle-high	326	703	85.5 (83.1, 87.9)	5	82.5 (77.9, 86.5)	69.4 (65.9, 72.8)	1.6
High	387	715	81.8 (79.3, 84.2)	5	81.7 (77.4, 85.4)	66.4 (62.8, 69.9)	1.5
*P* value for AUC comparison < 0.001							

SES, Socio-economic status; TP, true positive; FN, false negative; FP, false positive; TN, true negative; Sen., sensitivity; Spe., specificity; CI, confidence interval; AUC, area under curve.

 The discriminative ability of BIP to discriminate obese individuals from participants with overweight or normal weight did not correlate with age, education or ethnicity. There was no statistically significant difference between the estimated AUC for different subgroups regarding age, education and ethnicity in either gender (Table 5).

**Table 5:** Discrimination of Obesity from Overweight and Normal BMI According to Age Groups, Educational Level and Ethnicity Divided by Gender

**Gender/SES**	**TP+FN**	**FP+TN**	**AUC% (95% CI)**	**Cut**	**Sen.% (95% CI)**	**Spe. % (95% CI)**	**Likelihood Ratio+**
**Male**							
Age group							
< 50 years	200	1,729	88.2 (86.1, 90.2)	5	74.5 (67.9, 80.4)	86.1 (84.3, 87.7)	2.4
50-59 years	129	1,222	87.9 (85.1, 90.7)	5	83.7 (76.2, 89.6)	81.3 (79, 83.4)	2.1
59 + years	75	910	90.3 (86.5, 94.1)	5	88 (78.4, 94.4)	84.2 (81.6, 86.5)	2.6
*P* value for AUC comparison = 0.567							
Education							
Illiterate	91	1243	89.6 (86.0, 93.3)	5	84.6 (75.5, 91.3)	86.6 (84.5, 88.4)	2.4
Up to Diploma	204	1788	87.9 (85.9, 90.0)	5	78.4 (72.2, 83.9)	83.3 (81.5, 85.0)	2.3
Diploma & more	109	826	87.3 (84.2, 90.4)	5	78.9 (70.0, 86.1)	82.2 (79.4, 84.8)	2.1
*P* value for AUC comparison = 0.616							
Ethnicity							
Persian	255	2,105	89.5 (87.8, 91.2)	5	82.0 (76.7, 86.5)	84.9 (83.3, 86.4)	2.6
Turk	119	1,575	87.6 (84.5, 90.7)	5	79.8 (71.5, 86.6)	82.5 (80.5, 84.3)	2.4
Others	30	181	85.0 (78.0, 92.0)	5	63.3 (43.9, 80.1)	88.4 (82.8, 92.7)	2.7
*P* value for AUC comparison = 0.311							
**Female**							
Age group							
< 50 years	653	1,622	85.0 (83.4, 86.7)	5	83.2 (80.1, 85.9)	70.3 (68.1, 72.6)	1.6
50-59 years	393	1,056	86.9 (85.0, 88.9)	5	84.5 (80.5, 87.9)	74.2 (71.5, 76.9)	1.8
59 + years	270	973	88.3 (86.0, 90.5)	5	80.4 (75.1, 84.9)	81.3 (78.7, 83.7)	2.4
*P* value for AUC comparison = 0.063							
Education							
Illiterate	771	2418	87.2 (85.9, 88.6)	5	82.4 (79.5, 85.0)	76.8 (75.1, 78.5)	2.0
Up to Diploma	520	819	84.9 (83.1, 86.8)	5	83.7 (80.2, 86.7)	69.5 (66.8, 72.1)	1.6
Diploma & more	24	53	88.1 (81.0, 95.2)	5	87.5 (67.6, 97.3)	73.6 (59.7, 84.7)	1.7
*P* value for AUC comparison = 0.136							
Ethnicity							
Fars	833	2,003	86.5 (85.2, 87.9)	5	83.0 (80.2, 85.5)	74.0 (72.1, 76.0)	1.8
Turk	409	1,482	86.0 (84.1, 87.9)	5	82.2 (78.1, 85.7)	74.8 (72.5, 77.0)	1.9
Others	74	166	89.8 (85.9, 93.7)	5	87.8 (78.2, 94.3)	75.3 (68.0, 81.7)	1.9
*P* value for AUC comparison = 0.223							

SES, Socio-economic status; TP, true positive; FN, false negative; FP, false positive; TN, true negative; Sen., sensitivity; Spe., specificity; CI, confidence interval; AUC, area under curve.

## Discussion

 Approximately 60% of the study participants were obese or overweight. The study showed that BIP scores of 4 and 5 have acceptable sensitivity and specificity to discriminate normal weights from overweight/obese, and obese people from people with overweight/normal weight. We showed that gender, SES and ethnicity may be significantly associated with the discriminative ability of the BIP. The study results showed that age group and educational level were not associated with the validity of the BIP.

 In case of the discriminative ability of a BIP of 4, as the most appropriate identified cut-point to discriminate participants with normal BIM from overweight/obese participants, the highest AUC was observed among females from middle-high and high SES categories. This finding may be a result of the association of SES and body perception which has been reported previously.^18^ Accordingly, using the BIP among these populations may results in more accurate categorization of participants regarding their overweight status. This finding does not contradict the previous evidence on the overall validity of the BIP published from China and Iran.^10,19^ However, observed differences in the sensitivity and specificity of BIP across SES subgroups may lead to biased results induced by the resultant differential misclassification. In males, the discriminative ability of BIP was not different across SES categories, and therefore, using the BIP may result in more reliable findings when investigating the effect of SES on the epidemiology of overweight.

 In case of the discriminative ability of a BIP of 5, as the most appropriate identified cut-point to discriminate participants with obesity from normal weight/overweight, the lowest and highest AUC were observed among females from high and low SES categories. It may be a result of higher prevalence of obesity among Iranian females who belong to higher SES,^20^ and the notion that obese women perceive their body to be thinner than their actual body size.^21^ However, this finding reveals that the self-reported BIP by females from high SES may have lower validity to determine their obesity status. Accordingly, differential misclassification is possible when we are focused on the obesity across SES categories among females. On the other hand, an estimated AUC of around 90% reveals that the use of BIP among females from low SES may be an acceptable alternative method to determine their obesity.

 Another study finding is the significance of differences between the discriminative ability of the BIP across different ethnicities in both genders. This finding may be considered in line with available evidence on the determinants of body image dissatisfaction. There are several reports on the effect of ethnicity and race on body perception and dissatisfaction.^22,23^ Participants from Turk ethnicity had the lowest AUC, but it was acceptable and more than 80% in both males and females.

 Based on the study finding, BIP scores of 4 and 5 were the most appropriate cut-point to discriminate normal weights from the overweight/obese, and obese people from people with overweight/normal weight, respectively. These cut-points had the highest discriminative ability, sensitivity and specificity. This finding is in concordance with the results reported from Iran,^10,24^ but are not similar to cut-points recommended by Maruf et al.^25^ Accordingly, it seems that even if we can accept the validity of the BIP to discriminate BMI categories, the remaining question is what cut-points should be used in different populations. It may be helpful to determine the most appropriate cut-points before using BIP as a measure of overweight/obesity.

 A number of limitations need to be addressed. First, we used a cross-sectional design. Therefore, the study findings are valid to discriminate the current weight categories and may not be fully generalizable to the validity of the retrospective use of the BIP. In addition, as our sample population consist of adults aged 40 to 75 years, generalizing the study finding to younger ages may not be defensible. Also, it may be helpful to clarify that using BIP would be surely less precise than using BMI, but it may be considered as an alternative measure in the settings where measurement of BMI is not applicable. The study also showed that the difference between the cut-off point that discriminates normal and overweight/obese individuals and the cut-off point that discriminates obese and overweight/normal individuals was only one score (score four vs score five). This may suggest wide individual variability in selection of the figure. However, due to the stability of these cut-points across different subgroups, we believe that despite possible variability, these cut-points are valid to discriminate individuals with overweigh from individuals with normal weight, and again overweight people from those who are obese. On the other hand, the mentioned variability may also be possible when overweight/obese people are classified based on the precise value of BMI.

 In conclusion, although BIP may be a valid method to determine overweight and obesity in the general adult population, its validity depends on SES and ethnicity. It may be useful to use it in restricted homogenous populations.
